# Time to recovery from severe acute malnutrition and its predictors among children aged 6–59 months at Asosa general hospital, Northwest Ethiopia. A retrospective follow up study

**DOI:** 10.1371/journal.pone.0272930

**Published:** 2022-08-12

**Authors:** Fassikaw Kebede Bizuneh, Tadesse Tolossa, Nemera Eticha Bekonjo, Bizuneh Wakuma

**Affiliations:** 1 Department of Epidemiology, Faculty of Public Health, Institute of Health, Jimma University, Jimma, Ethiopia; 2 Department of Public Health, Institute of Health Sciences, Wollega University, Nekemte, Ethiopia; 3 Pawi General Hospital, Asosa, Ethiopia; 4 Department of Pediatrics and Neonatal Nursing, Institute of Health Sciences, Wollega University, Nekemte, Ethiopia; Dr Baba Saheb Ambedkar Medical College and Hospital, INDIA

## Abstract

**Background:**

Severe Acute Malnutrition (SAM) has become a major public health challenge in developing countries including Ethiopia, especially among the underprivileged population. Ethiopia is among the developing countries with the highest burden of acute malnutrition among under-five children. Though, plenty of studies were done on the magnitude of acute malnutrition among under-five children in Ethiopia, there is a limited evidence on time to recovery from SAM and its predictors among children aged 6–59 months in Ethiopia, particularly in the study area.

**Objectives:**

The study was aimed to assess the time to recovery from SAM and its predictors among children aged 6–59 months at Asosa general hospital (AGH), Benishangul Gumuz, Ethiopia.

**Methods:**

A Five years retrospective follow-up study design was employed among 454 children admitted with SAM in AGH from January 2015 to December 2019. The data were extracted from the patient medical records using checklist. The data were coded and entered into Epi-Data 3.1; then exported to STATA/SE-14 for analysis. Proportional Cox regression was performed to identify predictors of recovery time. A proportional hazard assumption was checked. Variables with AHR at 95% CI and P-value less than 0.05 in the multivariable Cox proportional regression was considered as significant predictors of recovery time.

**Findings:**

Among the 454 included records of children with SAM, 65.4% (95%CI: 50.1, 69.2) of them were recovered at the end of the follow-up with a median recovery time of 15 IQR(11–18)days. The incidence rate of recovery was 5.28 per 100 child days’ observations. Being HIV Negative (AHR = 2.19: 95% CI 1.28, 3.73), Marasmic (AHR = 1.69: 95% CI 1.18, 2.42), and marasmic-kwashiorkor child (AHR = 1.60: 95% CI (1.09, 2.37) independently predicted recovery time.

**Conclusions:**

Though the time to recovery from severe acute malnutrition was in the acceptable range, the proportion of recovery was found to be low in the study area compared to sphere standard. The prognosis of children with severe acute malnutrition was determined by the HIV status of the child and the type of malnutrition experienced. Further strengthening of malnutrition therapeutic centers and routine checkup of the nutritional status of HIV positive children should be emphasized to reduce child mortality and morbidity from under-nutrition.

## Introduction

Childhood malnutrition is still a major global health problem, leading to morbidity, mortality, and disability [1, 2]. It refers to a combination of nutritional disorders that include underweight, wasting, stunting, and micronutrient deficiency [3, 4]. Wasting is acute malnutrition due to a recent failure to receive adequate nutrition and may be affected by recent episodes of diarrhea and other acute illnesses [3]. Around 45% -60% of deaths for children less than 5 years of age were linked to under-nutrition, while this is mostly occurring in low- and middle-income countries [1, 2].

The current burden of malnutrition globally is unacceptably high and every country in the world is affected by malnutrition. In 2019, 144 million under-five children were suffering from stunting while 47 million were wasted of which 14.3 million were severely wasted worldwide [5]. Across the globe, SAM has contributed to 3.6 million under-five children death [2]. Severe acute malnutrition is the top 3 killer of under-five children next to pneumonia and neonatal sepsis with 20% of pediatric hospital admissions in Ethiopia and it is a reason for 25%- 30% of death in many poor countries [6, 7]. The problem of SAM is not only a medical disorder rather it is also a social disorder. Therefore, the successful management of severely malnourished patients requires both medical and social efforts [7].

The proportion of recovery from severe acute malnutrition among children aged 6–59 months in Ethiopia ranges from 58.4% [7] to 87% [8]. As per the international sphere standard recommendation, the recovery time of children admitted to stabilization center should be less than one month [9]. However, literature has showed that longer recovery time varying from 11 days to 8.7 weeks in Ethiopia [10–12]. Furthermore, according to the standard, the expected proportion of recovered children from SAM should exceed 75%. With this regard, existing pieces of evidence are showing as Ethiopia has failed to fully meet this standard. For instance, the proportion of children recovered from SAM ranges from 43.6% to 87% [13, 14]. Several factors such as age of the child, Tuberculosis infection, retroviral infection, type of malnutrition, and inpatient complications could influence both recovery rate and time from SAM [14–18]. Recovery from SAM remains challenging [3], insufficient [7], and even little is known about recovery time from SAM and its predictors among children aged 6–59 months in Ethiopia in general, and in the study area in particular. Therefore, this study aimed to determine the time to recovery from SAM and its predictors among 6–59 months old children treated in the stabilization center of Asosa general hospital.

## Methods

### Study area and period

The study was conducted in Asosa general hospitals in Benishangul Gumuz region, North West Ethiopia. The study period was from January 1^st^, 2015 to December 30^th^, 2019. The period was selected due to the highest malnutrition cases during the mentioned years. The data were abstracted from January 15, 2020 to February 15, 2020 from medical records of the child.

#### Study design

A hospital based retrospective follow up study design was employed.

### Populations

The source populations were all children aged 6–59 months admitted to TFC (therapeutic feeding center) for treatment of SAM in Asosa general hospital. The study population includes all eligible children with SAM admitted to SC (Stabilization center) from January 1^st^, 2015 to December 30^th^, 2019. All records of 6–59 months old children with SAM admitted to stabilization center of Asosa General Hospital from January 1^st^, 2015 to December 30^th^, 2019 were included in this study. However, incomplete records that missed socio-demographic information comorbidities; routine medications, and patient treatment outcomes (i.e. cure, death, not recovered and defaulter) were excluded.

### Sample size determination and sampling procedure

To determine the representativeness of the population, the sample size was estimated using the double population proportion formula in EPI INFO version 7 by considering different significant variables from the previous study, and by considering the following assumptions; the level of significance was 5% and the power 80% P1 = Proportion of recovery among exposed and P2 = Proportion of recovery among the non-exposed group [19]. The ratio of the population exposed to non-exposed was 1:1. Variables such as sex of the children, comorbidity, dehydration, and folic acid supplementation were used to calculate the sample size. Finally, the variable sex of children was selected for final sample size estimation which gave a sample size of 378.

The total admitted and treated children in Asosa General Hospital for SAM from January 1^st^, 2015 to December 30^th^, 2019 were 502. However, we included 454 medical records of children after excluding 48 incomplete records. Therefore, in this study no sampling procedure was used.

### Study variables

#### Dependent variable

The dependent variable of this study was time to recovery from severe acute malnutrition. The time was defined as the duration between the time children were diagnosed with SAM to the time the child recovered/discharged and it was estimated in days.

#### Independent variables

These include socio-demographic characteristics such as the age of the child, sex of the child, residence; comorbidities, types of comorbidity, and routine medications.

### Measurement

#### Survival time

Is the time in days from the child diagnosed with SAM to the occurrence of the outcome (recovered/censored).

#### Event (recovered)

Is a recovery of children from SAM or when the children fulfilled the discharge criteria determined by the ward physician.

#### Censored

Was those children who have not developed an event or those children who were not recovered from SAM (Death, defaulter, non-responder, transferred-out) at the end of the follow-up period. Death is when the child dies while on treatment for SAM, and the documented death was confirmed by the physician. Defaulter is when the child absents for two consecutive days. Transferred out is when the child moved to another health facility for further medical care.

#### Kwashiorkor

Is a severe form of undernutrition or malnutrition in children resulting from a diet excessively high in carbohydrates and low proteins.

#### Marasmus

This is a severe form of acute malnutrition characterized by emaciated physical appearance/severe wasting. It is a problem of carbohydrate deficiency.

#### Marasmus-kwashiorkor

Is a mixture of both kwashiorkor and marasmus. It is a problem of both carbohydrate and protein-containing food deficiency.

#### Co-morbidity

Any disease condition (acute or chronic) present at admission in addition to SAM which includes pneumonia, tuberculosis, diarrhea, anemia, vomiting, and retroviral infection.

### Data collection instrument and quality controls

A structured data abstraction tool was used for data collection purposes. The data abstraction tools were adopted from the Ethiopian federal ministry of health [20] updated SAM management guideline in 2013 with medical history sheet and related relevant published study. Three diploma nurses as data collectors and one public health officer supervisor were recruited. The training was given for both data collectors and supervisors for one day before data collection begun. For the quality of the data collection process and its completeness, the supervisor was following the overall data collection process.

### Data processing and analysis

Epi-data version 3.2 was used for data entry, and then the data was exported to STATA version 14 for further analysis. Before analysis, data were cleaned, edited by using simple frequencies and cross tabulation. Days were used as time scale to calculate median time to recovery from SAM. Descriptive non-parametric survival analysis such as Kaplan-Meier survival estimator and log-rank tests were used to estimate median recovery time during the treatment period and to compare time to recovery between groups (Types of residence, HIV Status, Zinc Supplementation status, Types of malnutrition), respectively. A cox proportional hazards regression model was used to determine factors associated with time to recovery from SAM. Factors associated with outcome variable at p-value < 0.25 in bivariable cox regression were selected for multivariable cox regression analysis. Adjusted Hazard Ratios (AHR) with 95% confidence intervals was computed and statistical significance was declared when it is significant at 5% level (p-value < 0.05).

The multivariable hazard ratio is the probability that an individual under observation experiences the event in a period centered on that point in time. Equation of Cox model is written as:

$$h\left(t\right)=h_{0}\left(t\right)\times exp\{b_{1}x_{1}+b_{2}x_{2}+\cdots +b_{p}x_{p}\}$$

Where the hazard function *h*(*t*) is dependent on a number of *p* covariates (*x*_1_, *x*_2_, …, *x*_*p*_), whose impact is measured by the size of the respective *coefficients* (*b*_1_, *b*_2_, …, *b*_*p*_). The term *h*_0_ is called the baseline hazard (constatnt HR), and is the value of the hazard if all the *x*_*i*_ are equal to zero (the quantity exp(0) equals 1). The ‘*t*’ in *h*(*t*) reminds us that the hazard may (and probably will) vary over time. An appealing feature of the Cox model is that the baseline hazard function is estimated non-parametrically (constant over a time), and so unlike most other statistical models, the survival times are not assumed to follow a particular statistical distribution [21].

**Fig 1:**
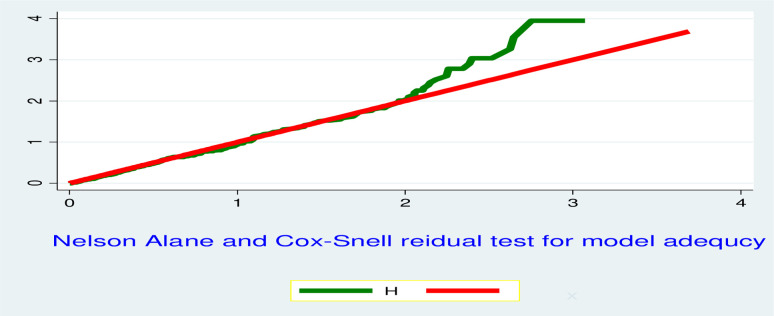
Final model adequacy graph based on Nelson Alan and Cox Snell residual test, 2020.

Cox–proportional hazard assumption was checked graphically (log-log plot) & statistically (Global goodness of fit test). Finally, Cox Snell residual test was used for checking final model adequacy (Fig 1).

#### Ethical clearance

Ethical clearance was obtained from the Ethical Review Committee of the College of Medicine and Health Sciences department of public health at Debre Markos University. Asosa general Hospital also rechecked for ethical compatibility and permitted data access. The ethics committee was aware that informed consents would not be obtained for this study as the study was conducted through a review of records.

## Result

### Baseline socio demographic and clinical characteristics

A total of 502 children were admitted to Asosa General Hospital from 2015 to 2019 with severe acute malnutrition. Of the total observed records, 48 were excluded due to unregistered outcomes and incomplete baseline data. Hence, 454 data were included for final analysis. The median age of children was 26.1 (SD ±17) months. From the eligible record of children, 250 (55.07%) were female, nearly one-third, 144 (31.72%) children were found in the age group greater than 36 months. More than three-fourth 348 (76.65%) of the study participants were from rural and pastoralist communities. Concerning comorbidities, diarrhea 282(50.22%) and pneumonia 282(50.22%) were the most observed comorbidity followed by vomiting 211(46.48%) whereas tuberculosis was the least observed comorbid condition next to HIV/AIDS among children (Table 1).

**Table 1:** Base-line socio-demographic characteristics and comorbidities of children admitted to Asosa general hospital, 2015–2019.

Variables	Category	Survival status		Total
		Recovered N (%)	Censored N (%)	
Age	6–11 months	52(17.5)	25(15.9)	77(16.9)
	12–23 months	93(31.3)	49(31.2)	142(31.2)
	24–35 months	58(19.5)	33(21.0)	91(20.0)
	≥36 months	94(31.6)	50(31.8)	144(31.7)
Sex	Male	129(43.4)	75(47.7)	204(44.9)
	Female	168(56.5)	82(52.2)	250(55.0)
Residence	Urban	84(28.2)	22(14.0)	106(23.3)
	Rural	213(71.7)	135(85.9)	348(76.6)
Co-morbidities				
Pneumonia	Yes	163(54.8)	65(41.4)	228(50.2)
	No	134(45.1)	92(58.6)	226(49.7)
HIV	Positive	15(5.0)	23(14.6)	38(8.3)
	Negative	282(94.9)	134(85.3)	416(91.6)
Anemia	Yes	100(33.6)	44(28.0)	144(31.7)
	No	197(66.3)	113(71.9)	310(68.2)
Diarrhea	Yes	142(47.8)	86(54.7)	228(50.2)
	No	155(52.1)	71(45.2)	226(49.7)
Tuberculosis	Yes	35(11.7)	19(12.1)	54(11.8)
	No	262(88.2)	138(87.9)	400(88.1)

### Children survival status and treatment outcomes

**Fig 2:**
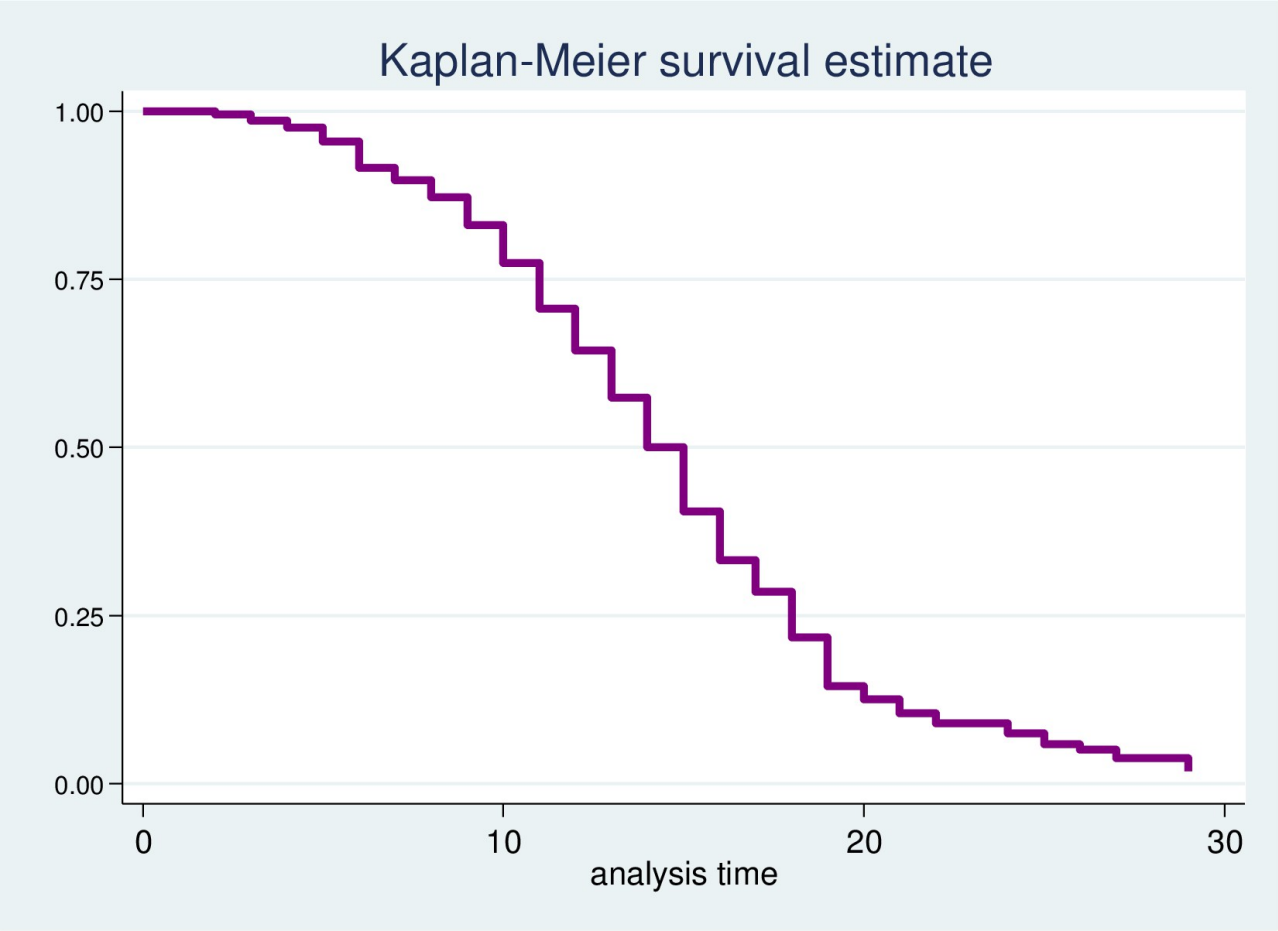
Kaplan-Meier survival function estimates of children with severe acute malnutrition at Asosa general hospital, 2015–2019.

A total of 454 children were followed for a mean time of 12.38 (SD±0.23) days. At the end of the follow-up time, 297(65.42%) observations were recovered. The patients had 2–29 days follow up times giving total 5625 person days observations of recovery free periods. Kaplan-Meier survival curve was used to estimate the survival status of children with SAM. The curve indicates that most children were recovered within the first twenty days (Fig 2). Marasmus (55.29%) was the predominantly observed type of malnutrition followed by marasmic-kwashiorkor (29.30%) and kwashiorkor (15.42%) respectively. At the end of the follow-up period, children were categorized as recovered/ cured (65.42%), defaulted (16.52%), died (11.45%), and transferred out (6.83%).

### Incidence and time to recovery

At the end of the follow-up period, 65.42% of observations were recovered, with an overall incidence rate of 5.28 per 100 (95% CI 4.71, 5.92) child days’ observation. The median time to recovery from severe acute malnutrition was 15 days (95% CI 14, 15). The highest incidence of recovery was observed at 15–20 days (20.06 per 100 child days’ observations) followed by 20–25 days (13.63 per 100 child days’ observations).

### Management protocol of SAM children

Management of admitted cases with severe acute malnutrition in the stabilization center was done as per the national guidelines. From the finding of this study, 357(78.43%) had given routine medication during inpatient treatment. Out of 228(50.22%) diarrhea cases, 220(96.6%) cases received zinc acetate (Table 2).

**Table 2:** Medication distribution for 6–59 months old children with SAM at Asosa general hospital, North-west Ethiopia (n = 454) 2015–2019.

Variables	Category	Survival status		Total N (%)
		Recovered N (%)	Censored N (%)	
Vitamin A	Yes	233(78.45)	133(84.71)	366(80.62)
	No	64(21.55)	24(15.29)	88(19.38)
Zinc supplementation	Yes	143(48.15)	77(49.04)	220(48.46)
	No	154(51.15)	80(50.96)	234(51.54)
Folic acid	Yes	217(73.06)	122(77.71)	339(74.67)
	No	80(2694)	35(22.29)	115(25.33)
Deworming	Yes	132(44.44)	61(38.85)	193(42.51)
	No	165(55.56)	96(61.15)	261(57.49)
Antibiotics	Yes	235(79.12)	122(77.71)	357(78.63)
	No	62(20.88)	35(22.29)	97(21.37)
F-75 formula milk	Yes	282(94.95)	148(94.27)	430(94.71)
	No	15(5.05)	9(5.73)	24(5.29)
F-100 formula milk	Yes	283(95.29)	79(50.32)	362(79.74)
	No	14(4.71)	78(49.68)	92(20.26)
Intravenous fluids	Yes	73(24.58)	32(20.38)	105(23.13)
	No	224(75.42)	125(79.62)	349(76.87)
Blood transfusion	Yes	62(20.88)	34(21.66)	96(21.15)
	No	235(79.12)	123(78.34)	358(78.85)

### Comparison of survival status

**Fig 3:**
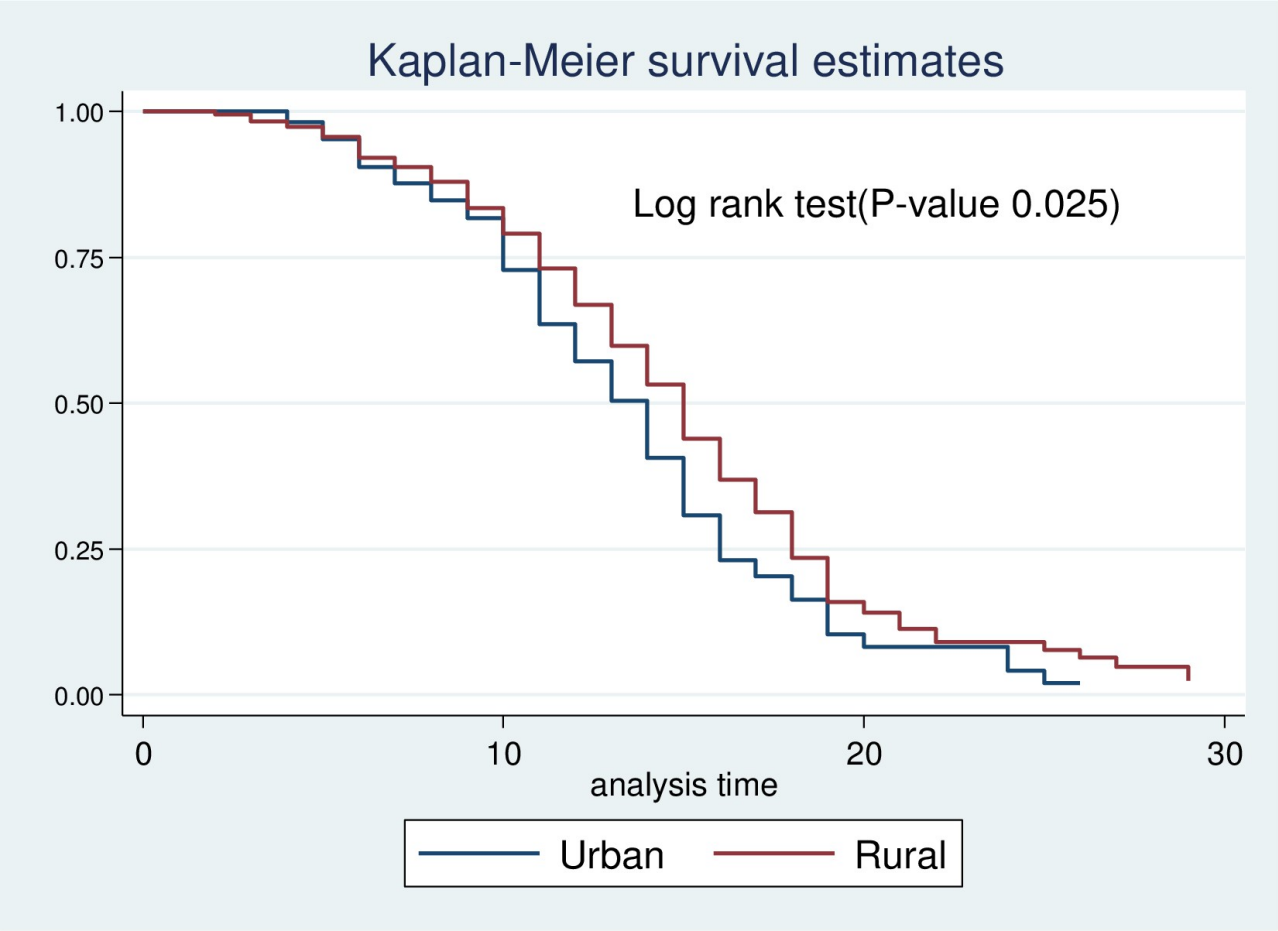
Log-rank survival estimate for time to recovery of SAM children by residence.

**Fig 4:**
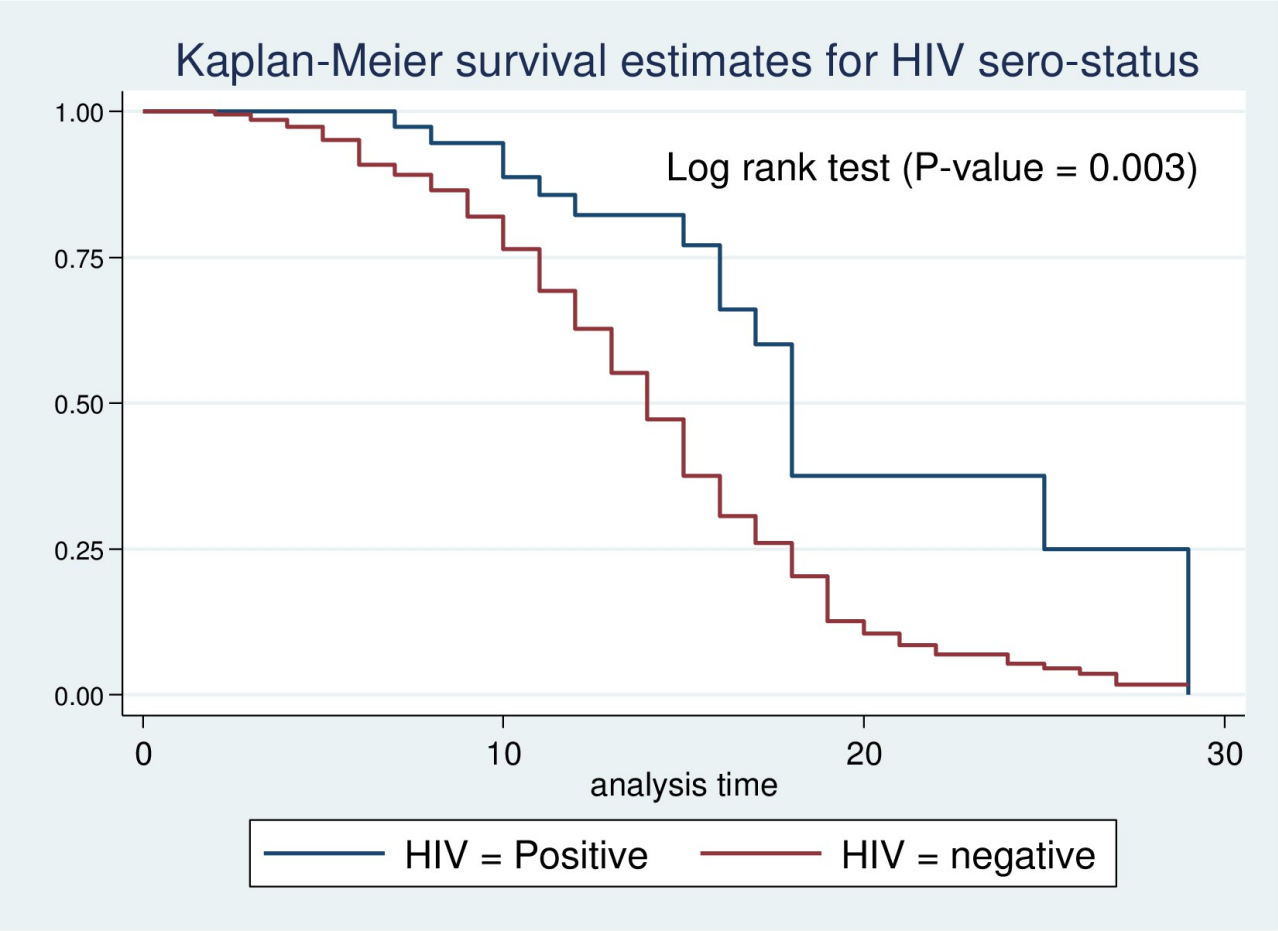
Log-rank survival estimate for time to recovery of SAM children by HIV status.

**Fig 5:**
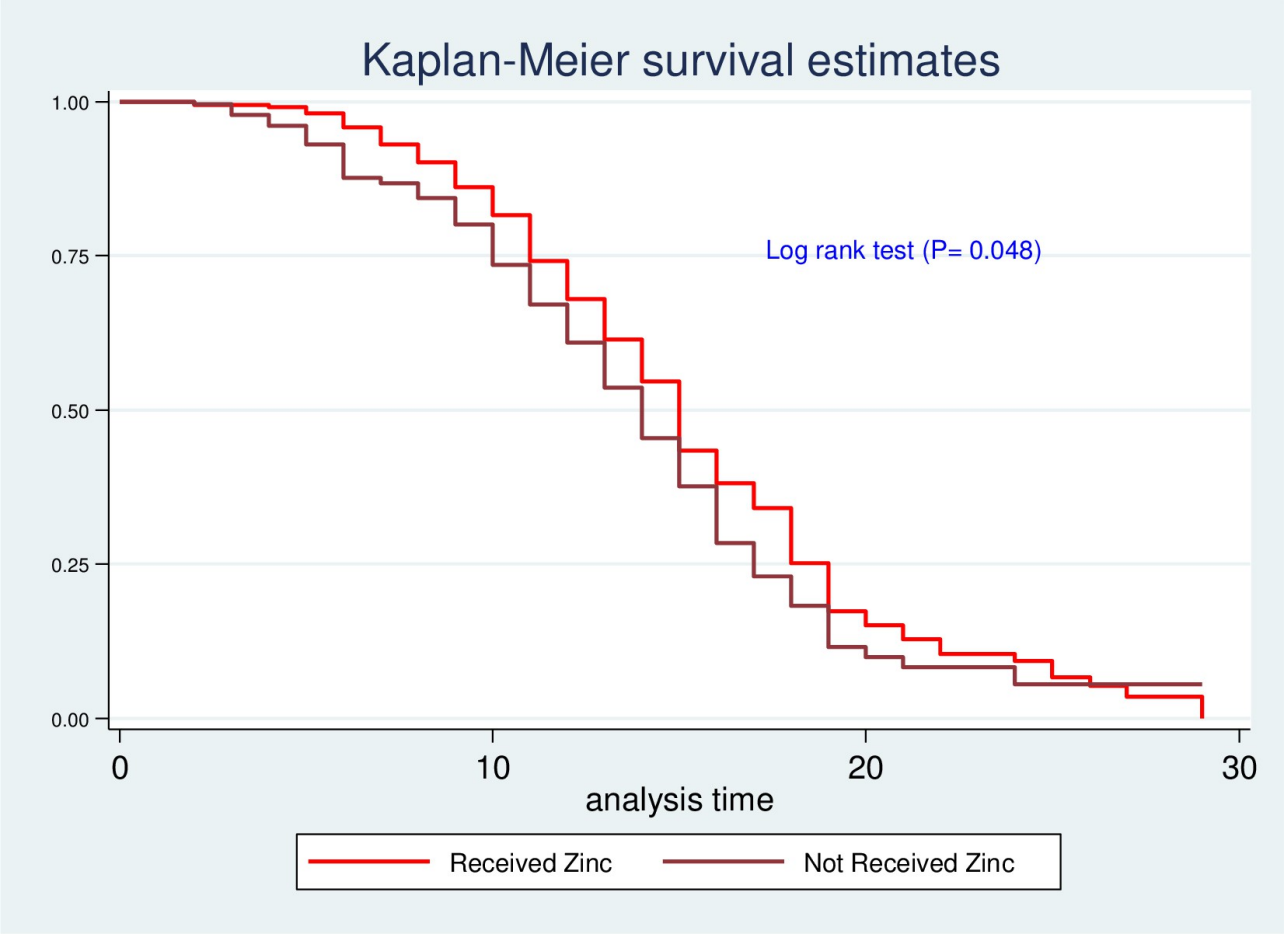
Log-rank survival estimate for time to recovery of SAM children by zinc supplementation.

**Fig 6:**
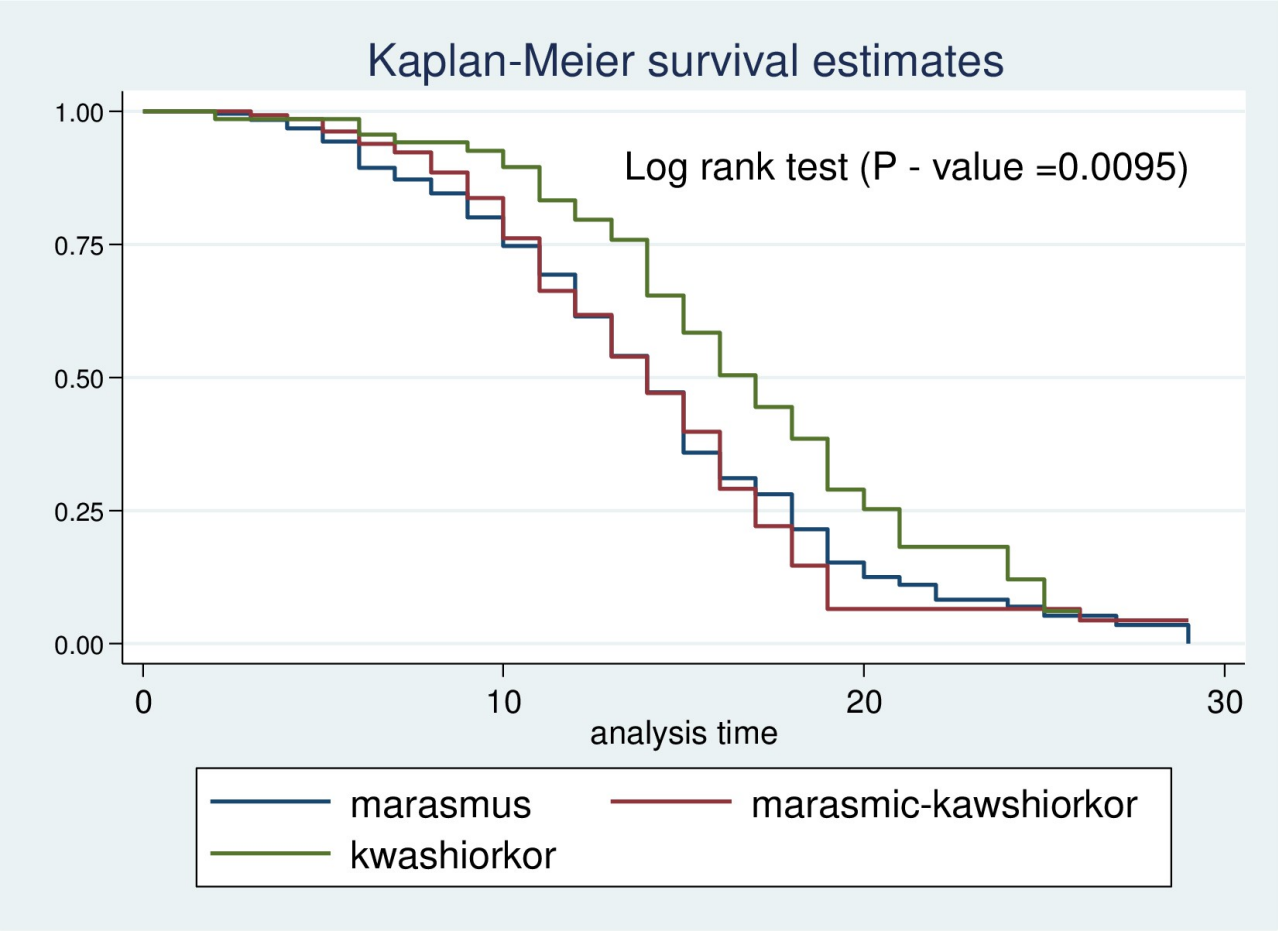
Log-rank survival estimate for time to recovery of SAM children by types of malnutrition.

Kaplan-Meier survival estimate was performed to compare survival probability between categories of different predictors. In addition, the significant difference in survival probability among predictors was evaluated by log-rank test. Accordingly, survival time among different predictors such as residence (Fig 3), HIV status (Fig 4), zinc supplementation (Fig 5), and types of malnutrition (Fig 6) was significantly different in survival time at 95% CI.

### Predictors of time to recovery from severe acute malnutrition

During bivariable analysis, 8 variables that have P-value <0.25 were selected for multivariable Cox regression (residence, malnutrition types, vomiting during admission, having NGT during admission, diarrhea during admission, Zinc acetate administration, routine antibiotic, HIV status). Three of the predictors were found to have a statistically significant association with time to recovery during multivariable cox proportional regression analysis at a 95% confidence level. This study found that children diagnosed with marasmus had 1.69 times early recovery as compared to those diagnosed with Kwashiorkor (AHR = 1.69, 95%, CI: 1.18–2.42). Similarly, children with marasmic kwashiorkor had 1.60 times recovered early compared to children with kwashiorkor (AHR: 1.60,95%, CI: 1.09–2.37). In addition, the chance of early recovery for HIV-negative children was two-fold higher compared to their HIV-positive counterparts (AHR: 2.19, 95%, CI: (1.28–3.73) (Table 3).

**Table 3:** Multivariable Cox-regression analysis predictors of time to recovery from SAM among 6–59 months old children at Asosa general hospital, 2015–2019.

Variables	Category	Survival status		CHR	AHR	P value
		Event N (%)	Censored N (%)			
Residence	Urban	84(28.28)	22(14.01)	1	1	
	Rural	213(7172)	135(85.99)	1.31(1.02–1.69)	0.79 (0.61–1.02)	0.075
Zinc supplementation	Yes	143(48.15)	77(49.04)	0.80 (0.64–1.01)	0.82(0.65–1.04)	0.109
	No	154(51.85)	80(50.96)	1	1	
Types malnutrition	Marasmus	163(54.88)	88(56.05)	1.60(1.13–2.29)	1.69(1.18–2.42)	0.004*
	Marasmic-Kwashiorkor	96(32.32)	37(23.57)	1.68(1.15–2.45)	1.60(1.09–2.37)	0.018*
	Kwashiorkor	38(12.79)	32(20.38)	1	1	
Vomiting	Yes	124(41.75)	87(55.41)	0.80(0.64–1.01)	0.88(0.69–1.11)	0.288
	No	173(58.25)	70(44.59)	1	1	
Diarrhea	Yes	142(47.81)	86(54.78)	1	1	
	No	155(52.19)	71(45.22)	1.19 (0.95–1.49)	1.07(0.5–1.35)	0.558
Antibiotics	Yes	235(79.12)	122(77.71)	1.09(0.85–1.36)	0.97(0.71–1.1)	0.821
	No	62(20.88)	35(22.29)	1	1	
NGT at admission	Yes	115(38.72)	72(45.86)	1	1	
	No	182(61.28)	85(54.14)	1.31(1.03–1.67)	1.11(0.87–1.40)	0.400
HIV status	Positive	15(5.05)	23(14.65)	1	1	
	Negative	282()94.95	134(85.35)	1.04(1.01–1.6)	2.19(1.28–3.73)	0.004*

CHR = Crude hazard ratio. AHR = Adjusted hazard ration, CI = confidence interval

## Discussion

The current study presented time to recovery from severe acute malnutrition and its predictors among children aged 6–59 months admitted to Asosa general hospital. This study found that the median time to recovery from severe acute malnutrition was 15 days. This is comparable with the study done in Pawi(14 days) [22], Gedeo(13 days) [23], Addis Ababa(17 days) [24]. However, the median time of recovery in this study is far faster than the median recovery time reported in Dire Dawa (61 days) [12], Arba Minch(49 days) [16], and Southern Ethiopia(26 days) [15] and a bit faster than recovery time reported in Jimma(19 days) [18]. However, it is a bit longer duration compared to recovery time reported in the Amhara region [10, 11].

The present study found an overall incidence rate of 5.28 per 100 child day’s observation with the highest incidence of recovery during 15–20 days((20.06 per 100 child days’ follow-up) followed by 20–25 days(13.63 per 100 child days’ observations). This reduced incidence of recovery with increased duration of hospital stay might be attributable to inpatient complications and increased risk of nosocomial infections. It is comparable with the study findings from Addis Ababa (4.6) [24], Southern Ethiopia (3.61) [15], Jimma (4.06) [18], East Amhara Hospitals(6.9) [10], Pawi, Benishangul Gumuz(5.3) [22]. This might be due to the relative similarity in readiness of health care facilities and socio economic characteristics of the study subjects. However, it is far lower compared to the study in Dire Dawa (17.23 child day’s observation) [12]. This might be due to the larger sample size in the study done at Dire Dawa.

The finding of this study has also indicated that the proportion of recovered children after treatment of SAM was 65.4% (95%CI: 50.12–69.24). This proportion is lower than the national and international sphere standard guidelines that recommend the recovery rate greater than 75% [9, 25]. However, it is in line with the study finding of seven health center in Gonder 65.3% [26], Nekemte Referral Hospital 66.8% [27] Southwest Ethiopia 67.7% [28], University of Gonder comprehensive specialized hospital)27l 69.2% [3], and in selected health institutions of Amhara region 62.13 [29]. Moreover, the finding is lower than the study finding from Yekatit 12 Hospital in Ethiopia 81.3% [30], SNNP of Ethiopia 87% [8], Addis Ababa, 79%, [24], East Amhara Hospitals 74.49% [10], Meta-analysis in Ethiopia 70.5% [31] and Shebedino, Southern Ethiopia 79.6% [32]. The discrepancy might be due to differences in socioeconomic status, and quality of health care provided to Children in the stabilization center. In this study, there is a higher mortality rate of 11.45%, which is incomparable with the standard sphere project [9]. The difference may be due to delays in seeking healthcare [3], medical complications, and mismanagement of SAM [6]. Mismanagement of SAM in a treatment center is the major challenge to increase the recovery rate [33].

Regarding Predictors of time to recovery from severe acute malnutrition, the present study revealed that a child with marasmus had faster recovery time compared to a child with kwashiorkor. Besides, children with marasmic kwashiorkor recovered early compared to those diagnosed with pure kwashiorkor. This is supported by existing pieces of evidence from the Amhara Region [29]. This might be due to marked physiological intracellular electrolyte disturbance. In addition, edema is associated with infectious disease serving as culture media for bacterial overgrowth [20]. Furthermore, co-morbidities have also influenced the recovery rate of children from SAM. Children with negative HIV serostatus had a two-fold higher early recovery rate from SAM than their HIV-positive counterparts. A possible explanation for this might be due to immuno-compromise related to HIV infection. In addition, diarrhea associated with HIV infection can also exacerbate the existing nutritional adequacy and further prolong recovery time from SAM.

### Strength and limitation of the study

Current study used secondary data in which we forced to exclude data with un-recorded outcome and this in turn can reduce adequacy of sample. Furthermore, the factors that are stated in this study are limited to the factors those were on record only.

## Conclusion

In general, in this study, the proportion of recovered children from severe acute malnutrition was low compared to the national and international sphere standard protocol. The median recovery time from SAM varies among types of protein-energy malnutrition and children’s HIV serostatus. Further strengthening malnutrition treatment centers should get due attention to prevent under-five suffering and death from acute undernutrition. Moreover, appropriate implementation of severe acute malnutrition standard treatment protocols and routine checkups for the nutritional status of HIV-positive children has paramount importance.

## Supporting information

S1 Data Severe acute malnutrition data set. (DTA)

S1 File Steps followed in final model building. (DOCX)

## Abbreviations

AHR: Adjusted hazard ratio
CHR: Crude hazard ratio
FMOH: Federal Ministry of health
HIV: Human immunodeficiency virus
NGT: Naso-gastric tube
RTUF: Ready to use therapeutic feeding
SAM: Severe acute malnutrition
SNNP: Southern nation and nationality peoples
TFC: Therapeutic feeding center
TFU: Therapeutic feeding unit
WHO: World health organization
