# Supplementary material for: Time to recovery from severe acute malnutrition and its predictors among children aged 6–59 months at Asosa general hospital, Northwest Ethiopia. A retrospective follow up study
Source: PLoS One. 2022 Aug 12;17(8):e0272930. doi: 10.1371/journal.pone.0272930 (PMC9374216; doi:10.1371/journal.pone.0272930)
Supplement: S1 File — (DOCX) [file pone.0272930.s002.docx]

**Model one**

**Model two**

**Model three**

**Model four**

**Model five**

**Model six**

**Model seven**

**Model eight**

**The whole Model**

**Final Model**
